# The stability issue of fractured rock mass slope under the influences of freeze–thaw cycle

**DOI:** 10.1038/s41598-024-56346-1

**Published:** 2024-03-07

**Authors:** Naifei Liu, Yinliang Yang, Ning Li, Shihao Liang, Hua Liu, Cheng Li

**Affiliations:** 1 School of Civil Engineering, Xi’an University of Architecture and Technology, Xi’an, 710055 Shaanxi China; 2 Key Laboratory of Mine Geological Hazards Mechanism and Control, Ministry of Natural Resources, Xi’an, 710054 Shaanxi China; 3 Institute of Geotechnical Engineering, Xi’an University of Technology, Xi’an, 710048 Shaanxi China; 4 Shaanxi Key Laboratory of Geotechnical and Underground Space Engineering, XAUAT, Xi’an, 710055 Shaanxi China

**Keywords:** Freeze–thaw cycle, Fractured rock mass slope, Failure mechanism, Classification of frozen rock slope, Stability evaluation, Hydrogeology, Civil engineering

## Abstract

Freeze–thaw failure of frozen rock slope often occurs during engineering construction and mining in cold area, which poses a great threat to engineering construction and people's life safety. The properties of rock mass in cold region will change with the periodic change of temperature, which makes it difficult to accurately evaluate the stability of slope under the action of freeze–thaw cycle by conventional methods. Based on field investigation and literature review, this paper discusses the characteristics of frozen rock mass and the failure mechanism of frozen rock slope, and gives the types and failure modes of frozen rock slope. Then, the research status of frozen rock slope is analyzed. It is pointed out that the failure of frozen rock slope is the result of thermo-hydro-mechanical (THM) coupling. It is considered that freeze–thaw cycle, rainfall infiltration and fracture propagation have significant effects on the stability of frozen rock slope, and numerical simulation is used to demonstrate. The research shows that the safety factor of frozen rock slope changes dynamically with the surface temperature, and the safety factor of slope decreases year by year with the increase of freeze–thaw cycles, and the fracture expansion will significantly reduce the safety factor. Based on the above knowledge, a time-varying evaluation method of frozen rock slope stability based on THM coupling theory is proposed. This paper can deepen scholars' understanding of rock fracture slope in cold area and promote related research work.

## Introduction

With the continuous promotion of China's western development strategy and the implementation of the Belt and Road Strategy, many water conservancy, transportation and mining projects have started construction in permafrost areas. Rock slopes containing fracture ice are often encountered in engineering construction and mining in permafrost areas. Under the action of freeze–thaw cycle, such rock slopes may have serious freeze–thaw disasters, causing huge losses to industrial and agricultural production, and even burying villages, destroying towns and mining facilities, endangering transportation and energy projects, and often producing secondary disasters^1,2^. Therefore, it is necessary to study the related problems of fractured rock slope under the influence of freeze–thaw cycle.

The freeze–thaw disaster of fractured rock slopes in permafrost areas is a result of the thermo-hydro-mechanical (THM) coupling in fractured rock, involving phase change between ice and water during freeze–thaw cycles^3^. Several scholars have conducted research on slope stability in cold regions subjected to freezing and thawing cycles. Based on field monitoring, numerical simulation, and laboratory testing, Shin et al.^4^ emphasized the significant impact of freeze–thaw effects on slope stability and developed an early-warning system for slopes in cold regions based on continuous monitoring data. Subramanian et al.^5^ proposed a slope stability assessment approach based on two-dimensional numerical modelling that considers freeze–thaw cycles and snow water infiltration. Cong et al.^6^ demonstrated that shallow failures of frozen slopes are related to frequent snowmelt infiltration using a three-dimensional numerical model. Zhang et al.^7^ studied the influence of freeze–thaw action on landslide stability in loess landslide zones and identified it as an important factor along with underlying slope characteristics affecting landslide development. Qin et al.^8^ established a multi-physical numerical model for soil slopes on reservoir banks and deduced the evolution law of slope safety factors under changing hydrothermal conditions along sliding surfaces during freeze–thaw processes. Chou et al.^9^ suggested that settlements in cold regions with large temperature variations are greater than those observed in regions with smaller temperature changes. In addition, Yang^10^, Subramanian^11^, and Xiang^12^ also investigated the mechanical properties of rock masses and changes of slope stability under freeze–thaw actions.

Current researches on slope engineering in cold region are mostly focused on soil subgrade or cut slope, and rarely take rock slope as the research object. Luo et al.^13^ studied the influence of rock deterioration on slope stability under the action of freeze–thaw cycle; Yang et al.^14^ proved that the F–T cycle and creep characteristics of rock mass have a negative influence on the long-term stability of slope engineering; Zhang et al.^15^ established an evaluation system for the stability of rock slope in cold areas and provided the classification standard. It should be noted that only Li et al.^16^, Qin et al.^17^ conducted researches on the damage and progressive failure characteristics of fractured rock slopes under freeze–thaw conditions. Despite the increasing attention paid to the frozen rock problem in recent years, the research has generally focused on mechanical testing of intact or fractured rock samples under the conditions of freezing or freeze–thaw cycles^18–20^. Only a few scholars have conducted research on rock slopes in cold regions, such as Wang^21^ and Huang^22^. It can be seen that the failure mechanism and failure mode of soil slope under the action of freeze–thaw cycle have been relatively mature. However, the understanding of fractured rock slope is still insufficient, and the related research results are rarely reported.

In view of the shortcomings of previous research, this paper takes fractured rock slopes encountered in engineering construction and mining in China as the research object to discuss the characteristics and stability of rock slopes under freeze–thaw action. In Section "Influence of freeze–thaw cycles on frozen rock slope", the failure mechanism and failure type of rock slope under freeze–thaw cycle are studied. In Section "Mechanical properties of frozen rock and classification of engineering slopes", the mechanical properties of frozen rock mass are analyzed and the classification of rock slope is given. Then, in Section "Research status of frozen rock slope", the research status of fractured rock slope in cold area are discussed. In Section "Stability evaluation of fractured rock slope in cold region", the stability evaluation method of rock slope in cold region considering THM coupling is proposed and demonstrated by numerical simulation. In the last section, some significant conclusions are drawn from this study.

## Influence of freeze–thaw cycles on frozen rock slope

Frozen rock slope refers to the slope where natural or man-made fractured rock mass and ice coexist. As a result of the periodic temperature changes, the rock mass of the slope is subjected to a reciprocating freeze–thaw cycle, which results in a different stability mode and failure mechanism than that of an ordinary slope. This section aims to analyze the influence of the freeze–thaw cycle on the stability of fractured rock mass slopes and the failure types of such slope.

### Factors affect the stability of frozen rock slope

The stability of the frozen rock mass slope is influenced not only by general factors such as rainfall or earthquakes but also by ice content, freezing-and-thawing action, and temperature changes^23,24^. Depending on the location of the frozen rock mass slope, it can be classified as a seasonally frozen rock slope (first-class slope) or a multi-year frozen rock slope (second-class slope).

The first-class slope is mainly affected by late autumn and early winter rain or snowfall and periodic temperature fluctuations. When the temperature decreases, rainfall infiltrates into fractures in the rock mass and freezes, resulting in significant frost-heaving pressure. This force leads to fracture extension followed by melting water movement, causing further expansion of fractures. Additionally, when the ice disappear as the weather warms in spring, the bonding strength of fractured rock mass significantly decreases which may lead to slope collapse^1^. K.Terzaghi^25^ also noted that surface freezing of high-dip angle rock mass slopes increases groundwater levels leading to an increase in hydraulic pressure within fracture-planes which induces slope failure.

**Figure 1:**
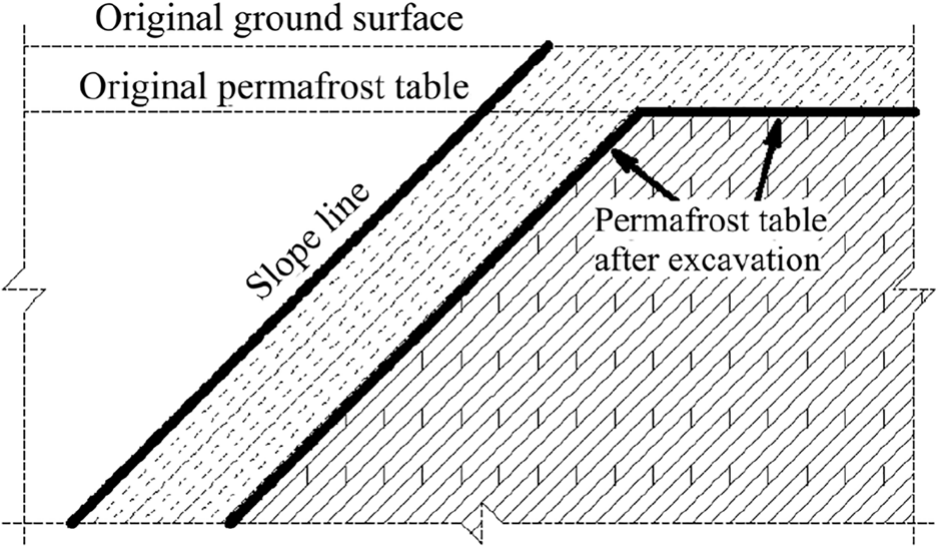
The effect of slope excavation on permafrost.

The second-class slope is formed in the construction of the projects in cold regions, which is influenced not only by the factors that affected the first-class rock slope but also by the perennially frozen rock mass. Construction exposes the rock mass that is not affected by climate, which not only changes the initial stress field of the rock mass, but also changes the temperature field and water field, resulting in the decrease of the permafrost table, as shown in Fig. 1.

It is also necessary to focus on the changes of rock physical and mechanical properties and the cumulative effects of freeze–thaw cycles on the stability of rock slopes in cold areas^26^. As open-pit construction is a long-term process, the stability of open-pit slope in cold areas is not only affected by conventional factors and periodic temperature, but also by engineering construction (construction dynamic load, etc.), which makes the stability analysis of frozen rock slope more complicated.

### Failure mechanism of frozen rock slope

**Figure 2:**
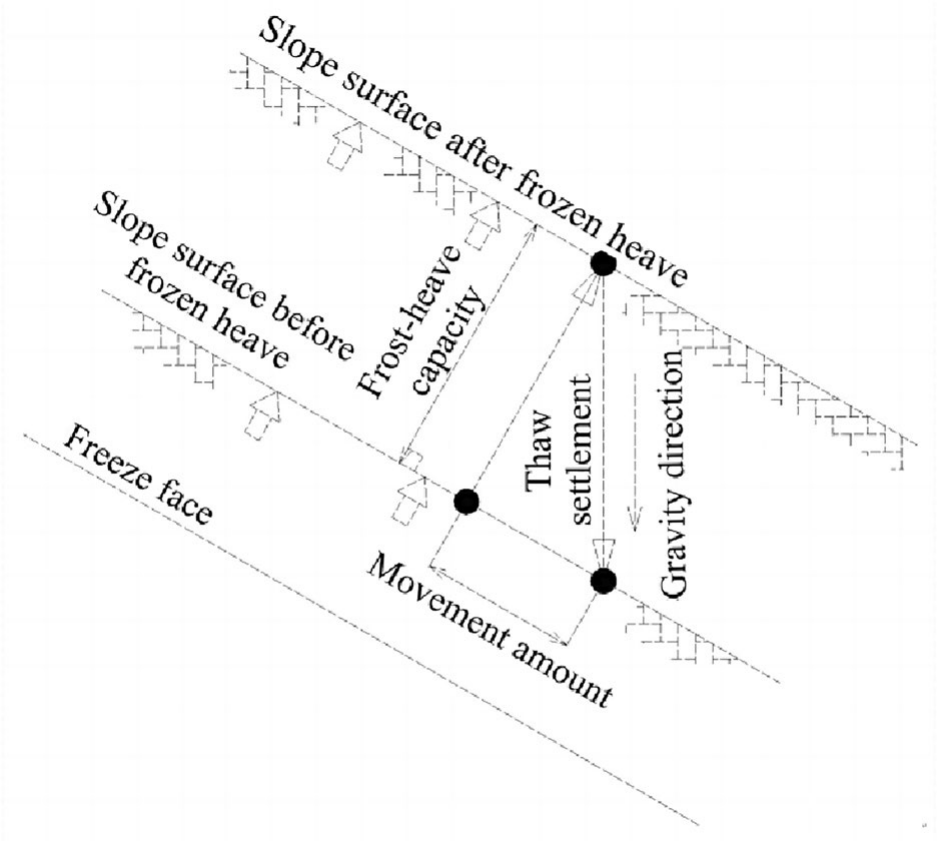
The freeze–thaw process of slope strata.

In cold areas, slope failure occurs mainly due to cyclic freeze–thaw caused by periodic temperature changes^26^. During the winter, the slope is covered with ice and snow. With the approach of spring, the ice and snow melt and penetrate into the lower soil^27^. At this point, the upper soil has melted, while the lower soil is still frozen, which means that water can no longer penetrate the lower strata. As a result, the strata at the upper end of the slope is saturated or supersaturated, reducing its shear strength to a great extent. Consequently, the freeze–thaw interface becomes a sliding surface that continuously creeps and deforms under repeated day and night air temperature and gravity action, resulting in the shallow slump of the slope^1^, as shown in Fig. 2.

**Figure 3:**
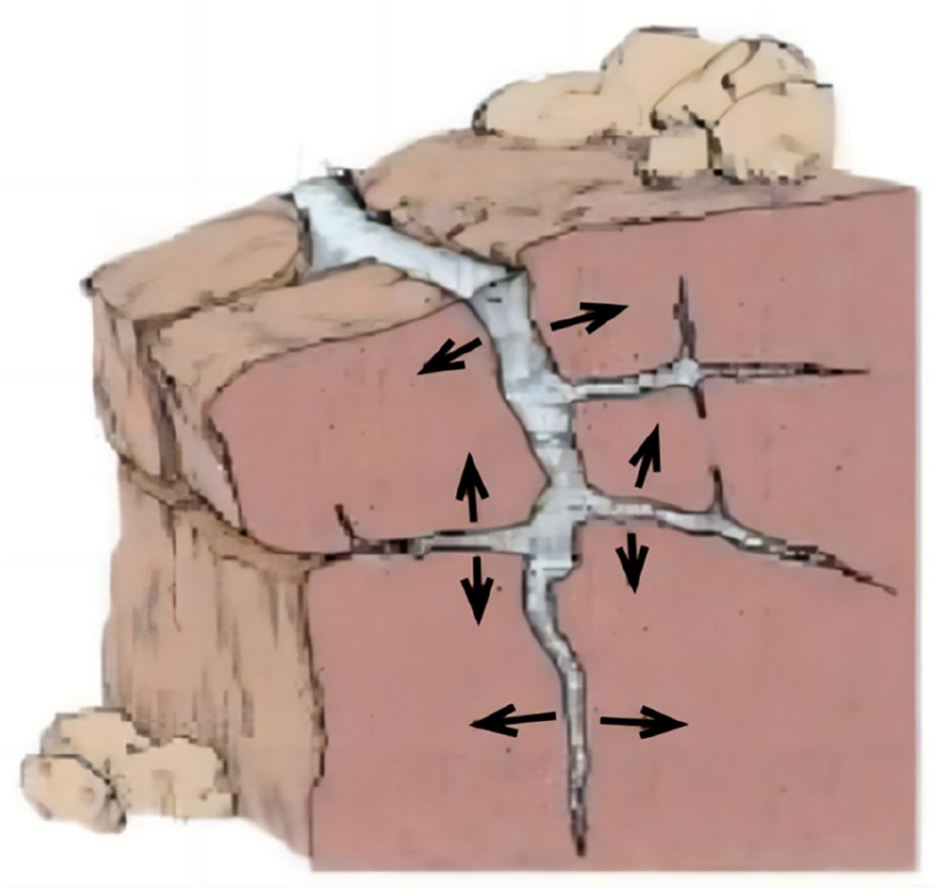
Sketches of freezing–thawing damage^3^.

There exists a notable difference between the failure mechanisms of frozen fractured rock slopes compared to those of frozen soil slopes. Due to fractures present in frozen rock slopes, their failure mechanism becomes more complex. Water inside fractured rock masses transforms into ice when its temperature reaches freezing point. After the water transforms into ice, its volume will expand by 9%^28^, which generates pressure (frost heaving force) on the fractured rocks, leading to fracture expansion as shown in Fig. 3. As temperatures rise again, melted water infiltrates newly formed fractures; subsequently refreezing occurs during temperature drops leading to further splitting of rocks. As a result, the strength of the rock mass will continue to decrease over time. The failure of frozen rock slope is mainly due to the periodic change of air temperature, which constantly destroys rock mass under the action of the reciprocating freeze–thaw cycle. In addition, the seasonal change and the temperature difference between the day and night cycle also have an important influence on the mechanical properties of rock mass, especially the rock mass with low strength and high water content.

Furthermore, some frozen rock slopes have loose overburden at a certain depth on the surface, which requires that the failure mechanism of frozen rock slopes also consider frozen soil slopes and their mutual influence.

### Failure types of frozen rock slope

Slope instability in cold regions is primarily caused by the freeze–thaw cycle, rather than rainfall or other external loads^27^. Based the actual investigation and theoretical research, it was becoming clear to failure types of the frozen soil slope. Mcroberts and Morgenstern^29^ have typed the failure types of frozen soil slope into the mudflow, landslip and collapse. According to Niu et al.^30^ unstable slopes in cold regions can be divided into normal frozen landslides, normal melting landslides, and freeze–thaw landslides depending on their causes. Shan et al.^31^ obtained that landslides are seasonal, progressive and low-angle.

Relatively fewer studies have been conducted on the classification of failure types in frozen rock slopes. Chen et al.^32^ divided the failure types of freeze–thaw collapse of the rock mass slope into the toppling type, the pusher type and various rock fall. Considering the division of the failure types of the frozen soil slope and the damage of the real frozen rock mass slope, the failure types of the frozen rock mass slope is divided into four types.

**Figure 4:**
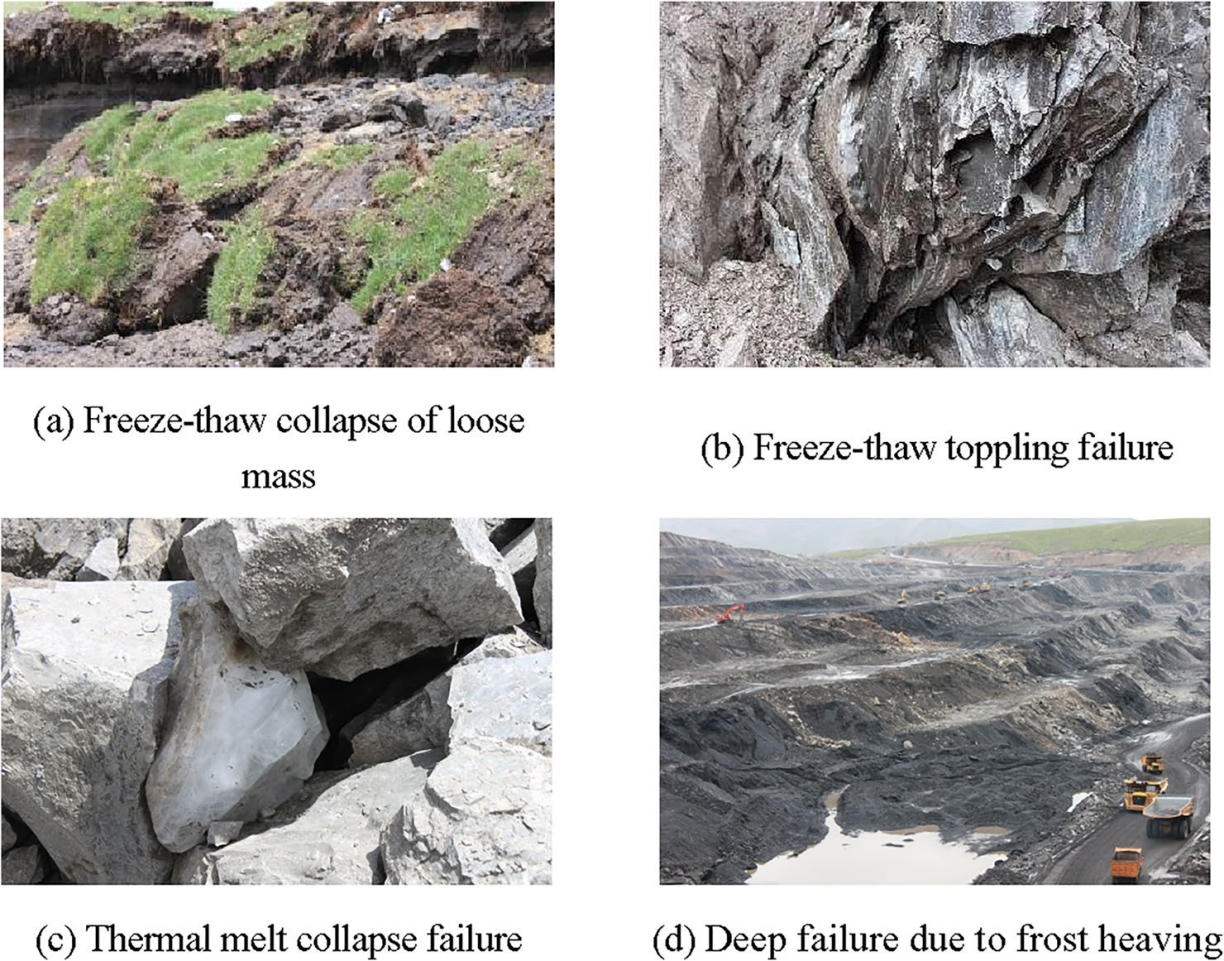
Failure types of frozen rock mass slope (All images are taken by the author).

1. Freeze–thaw collapse of loose mass:

The upper part of a rock slope covered with loose material exhibits similar properties to that of a frozen soil slope. Under the influence of freezing and thawing, the upper portion of a rock slope is often destroyed along the interface between rock and soil. This type of failure is commonly observed in open pit mining slopes in the Tibetan plateau (Fig. 4a).

(2) Freeze–thaw toppling failure:

In cold regions, it is common for toppling failures to occur on steep frozen rock slopes with significant weathering and sufficient groundwater recharge^33^. The immense frost heaving force generated by ice-water phase changes during winter can lead to such failures. Additionally, freeze–thaw cycles may cause toppling failures during other seasons as well, as depicted in Fig. 4b.

(3) Thermal melt collapse failure:

Freeze–thaw damage induces microstructural deterioration in frozen rock masses, particularly those with high water content. Under the influence of freeze–thaw cycles, thermal melt collapse represents the primary form of failure for frozen rock slopes, predominantly occurring in springtime. Winter frost heaving intensifies weathering within the rock mass; subsequently, rising temperatures transform ice into water during springtime, reducing bond strength between rocks and resulting in slope collapse and failure (Fig. 4c).

(4) Deep failure due to frost heaving.

Besides the above three failure types, the frozen rock mass slope might be destroyed along the structural surface or permafrost base in deep part due to the action of engineering disturbances and frost heaving (Fig. 4d).

## Mechanical properties of frozen rock and classification of engineering slopes

The fracture water freezes and melts repeatedly under the freeze and thaw cycles which make the frozen rock mass slope quite different from the frozen soil slope. Some scholars have studied the distribution and failure mode of slope in cold area^34^. On the basis of the deep analysis of the frozen rock mass properties as soon as considering the actual situation of the real rock slope, this paper divides the types of the frozen rock mass slope by reference to the soil slope.

### Characteristics of frozen fractured rock mass

1. Strong structural property.

Due to the seasonal changes in the air temperature and the alternation of day and night, the water within the rock mass fracture is continuously frozen and thawed, which causing the rock fracture to expand continuously. With the periodic change of air temperature, the physical and mechanical parameters of rock mass also deteriorate. Therefore, a large number of fractures appear in the frozen rock mass, which shows strong structural characteristics.

(2) Anisotropy.

The periodic change of temperature and the existence of temperature gradient cause the vertical migration of water in the fracture, which leads to the continuous growth of fracture ice and the continuous expansion of the fracture, so that the frozen rock shows significant anisotropy.

(3) Anisotropy.

In comparison to porous media, fractured rock mass exhibits significant differences. The movement of water from the bottom to the top occurs through capillary action under the influence of temperature in porous media. Most pores in fractured rock masses are closed and fracture openings are often large, the migration of water from the bottom to the top by capillary action is generally not significant. However, under the action of temperature gradient, water migration still exists in the fracture.

(4) Freeze–thaw damage characteristics.

Freezing can increase the strength of soil due to the transformation of water into ice. However, the freezing action makes fractured rock mass even weaker because of the existence of freeze–thaw damage. In low temperature, fracture water will turn into ice, and the resulting volume expansion will cause fracture expansion. Multiple freeze–thaw cycles will cause irreparable damage to rock mass.

### Classification of frozen rock slope

According to the occurrence state of the frozen rock mass and the failure mechanism of such slope, in this paper the types of the frozen rock mass slope is divided into the following 4 classes.

1. Frozen cataclasite slope.

**Figure 5:**
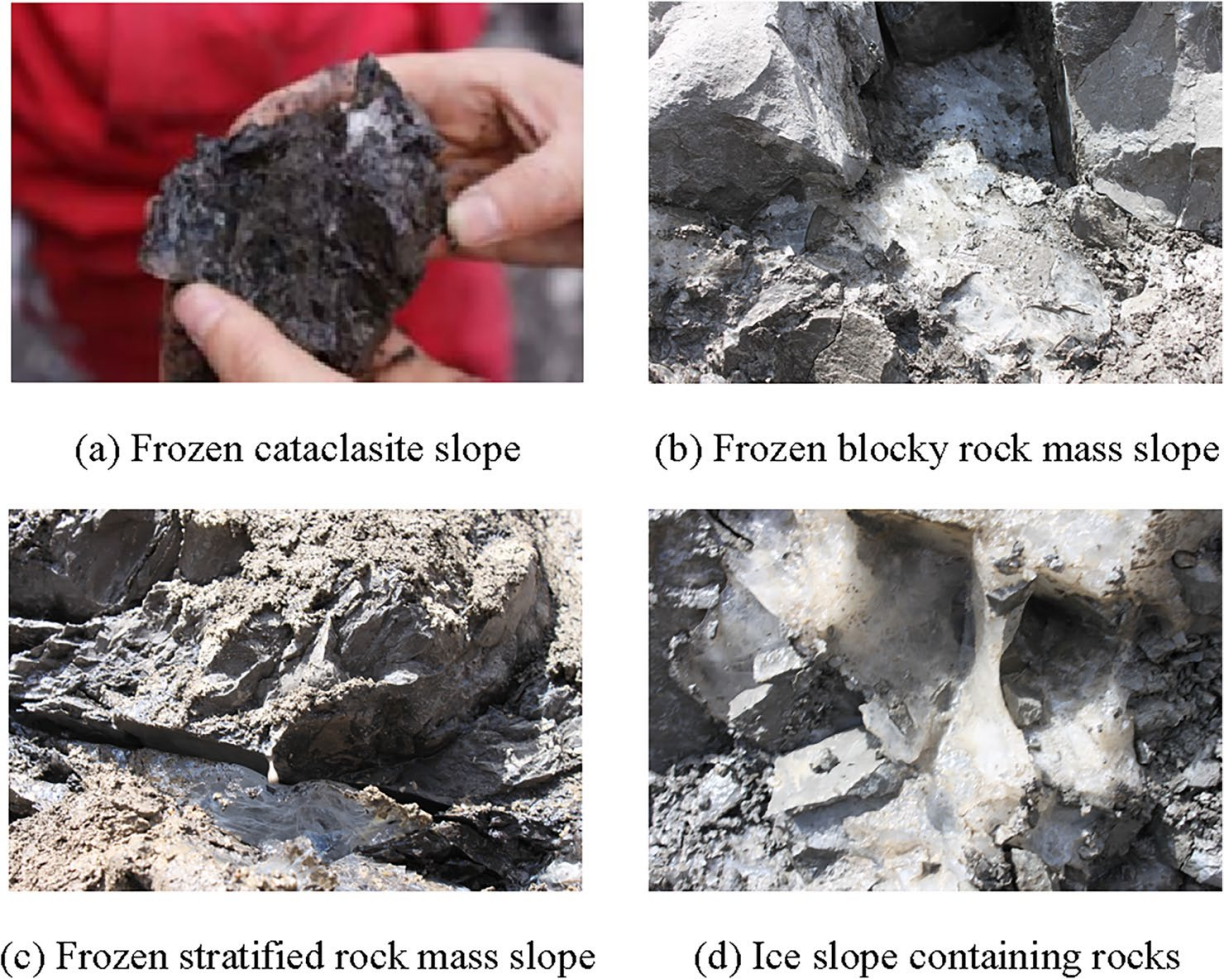
Classification photos of frozen rockmass slope (All images are taken by the author).

During the freeze–thaw cycle and under strong weathering conditions, the fractured rock mass becomes loose and fragmented. When there is an adequate water supply, the rock mass becomes saturated with frozen ice at low temperatures. The material is characterized by a loose rock mass after melting, with an ice content of less than 50% and approaching zero cohesion (Fig. 5a).

(2) Frozen blocky rock mass slope.

Compared to cataclastic frozen rock, this type of slope exhibits a lower degree of weathering. Weathering and freeze–thaw splitting have resulted in the distribution of the rock mass into blocks. In winter, the rock mass is surrounded by ice with an ice content of about 20%. Even when the ice melts, some cohesion remains within the rock mass (Fig. 5b).

(3) Frozen stratified rock mass slope.

This kind of slope usually develops in the banding rocks and the ice is extended in the rock layer. If there is enough water, the ice might even separate the rock layer completely. This kind of slope is developed in the permafrost regions (Fig. 5c).

(4) Ice slope containing rocks.

These slopes differ significantly from previous types as they primarily consist of ice with an ice content exceeding 50%. The rocks are entirely encased in ice. In this paper, we define slopes containing both rocks and an icy layer as “Ice Slopes Containing Rocks” when their volume ratio exceeds 1.0 (Fig. 5d).

## Research status of frozen rock slope

The frozen rock slope is constantly affected by the cyclic variable temperature load, which results in rock damage and strength decline^35^. Therefore, to accurately grasp the stability of the frozen rock slope under the influence of freeze–thaw cycle, the theoretical research should be strengthened, including the indoor and outdoor test research and the research on constitutive model.

### Laboratory test of frozen rock

The frozen rock specimen has typical brittle failure characteristics^36^. The freeze–thaw damage of porous rock is mainly induced by frost heave pressure, and its water saturation and mechanical properties are important for quantifying frost heave^37^. With the need of engineering construction, many scholars have carried out indoor test research on frozen rock mass. Mufundirwa et al.^38^ clearly observed the development of rock fractures. Zhang et al.^39,40^ obtained the total damage evolution equation and constitutive model of freeze–thaw rock under loads through the laboratory test. Tang et al.^41,42^ studied the macro- and meso-scale weakening behavior of S-RM under freeze–thaw cycles, revealing the strength degradation mechanism of S-RM after freeze–thaw cycles. Wang et al.^43^ revealed that the mechanical properties of granite would deteriorate with the increase of the number of freeze–thaw cycles. Only few scholars have paid attention to the role of fractures, and studied the failure mode, damage evolution law and physical parameter deterioration of fractured rock mass under freeze–thaw cycle. Huang et al.^44^ concluded that unidirectional freezing mode is more conducive to fracture propagation than uniform freezing mode. Zhao et al.^45^ studied the variation law of rock physical and mechanical parameters with the length of vertical fractures in the process of freeze–thaw cycles. Shen et al.^46^ conducted freeze–thaw cycle tests on single fractured sandstone samples with different fracture angles, and concluded that the fatigue failure strength under freeze–thaw action was proportional to the fracture angle. Luo et al.^47^ obtained the freeze–thaw damage degradation model of diabase through X-ray diffraction experiment. In order to fully understand the deformation and failure characteristics of frozen rock slope under the influence of freeze–thaw cycle, rock samples containing fractures should be selected to carry out systematic laboratory tests to study the relationship between the thermodynamic parameters of rock mass, such as:

1. The relationship between geometrical characteristics of fracture and thermal parameters such as thermal conductivity, heat capacity and thermal expansion coefficient.
2. The mechanical properties of rock samples with different fractures angles and widths under freeze–thaw cycles.
3. The THM coupling mechanism in fractured rock samples under different water content, temperature and stress conditions.

### Study on frozen rock constitutive model

**Figure 6:**
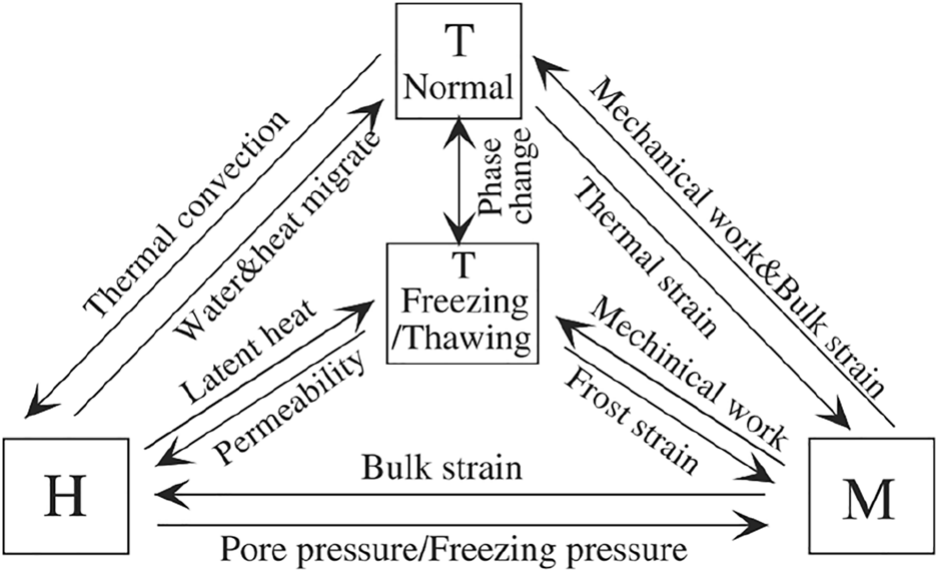
THM coupling relations under freeze–thaw cycle^3^.

In addition to helping engineers understand the degradation of performance of rock masses during freeze–thaw cycles, indoor test results cannot explain the fundamental relationship and interaction mechanism between thermo-hydro-mechanial within fractured rock masses in cold regions (Fig. 6)^48^. In light of this, it is necessary to strengthen research of the constitutive law of frozen rock mass.

The main problem in THM coupling study of frozen rock mass is that the rock mass is regarded as isotropic porous medium and the frozen soil mechanics theory is directly applied. Only a few scholars consider the effect of fracture when establishing the THM coupling model. Kang^48^ derived the THM coupling equation of fractured rock mass under frozen condition using the double-pore medium theory and considering the effect of ice-water phase transformation. Mu^49^, Liu^50^, Wang^51^, Huggel^52^ studies the variation law of rock thermal stability caused by air convection based on the principles of fracture mechanics. Hori and Morihiro^53^ simplified the internal pores of rocks into unconnected single fractures, and established a micro fracture mechanics model to describe the freeze–thaw process of rocks. Liu et al.^54,55^ derived the mass conservation equation, the balance equation, and the energy conservation equation of the frozen rock mass based on the irreversible thermodynamics theory, and established the THM model of the fractured rock mass in the cold region. Liu et al.^56^ considered the effect of temperature on the freezing rate, and adopted the equivalent thermal expansion coefficient method to simulate the frost heaving load, and established the single fracture frost heave model. Liu et al.^57^ developed a new ternary model to better reflect the nonlinear stress relaxation behavior of rocks under freeze–thaw conditions.

Although the effect of fractures has been considered in the above studies, the lack the consideration of the mechanism of hydrothermal migration in fractured rock mass under the action of freeze–thaw cycle still insists. Yang et al.^58^ verified that temperature gradient was the main driving force of water migration through sandstone experiments, but still regarded it as a porous medium. Murton et al.^59^ prepared limestone into 10 samples with different water replenishment conditions, which proves that the partial freezing resulted in the cracking of wet limestone blocks. Cheng et al.^60^ established a capillary-film moisture migration model of porous rocks, and gave the stress distribution, migration direction and migration path of the characteristic pores.

It can be seen that most studies are still in the stage of experimental exploration.The important manifestations of freeze–thaw damage of rock mass are fracture propagation and network evolution in rock mass. At present, there are few reports studying freeze–thaw damage of fractured rock mass from the perspective of water migration. Only a few scholars, such as Wang^61^, Yang^62^, have discussed the morphology and mechanism of water and heat migration in fracture under the influence of freeze–thaw. According to the current research situation and objective needs, the experimental research on the hydrothermal migration of fractured rock mass should be strengthened, so as to establish model of hydrothermal migration that fully considers the influence of fractures, and further embedded into the THM coupling model that can truly consider the effect of fractures, thus revealing the freeze–thaw characteristics of fractured rock mass.

### Field monitoring of frozen rock slope

**Figure 7:**
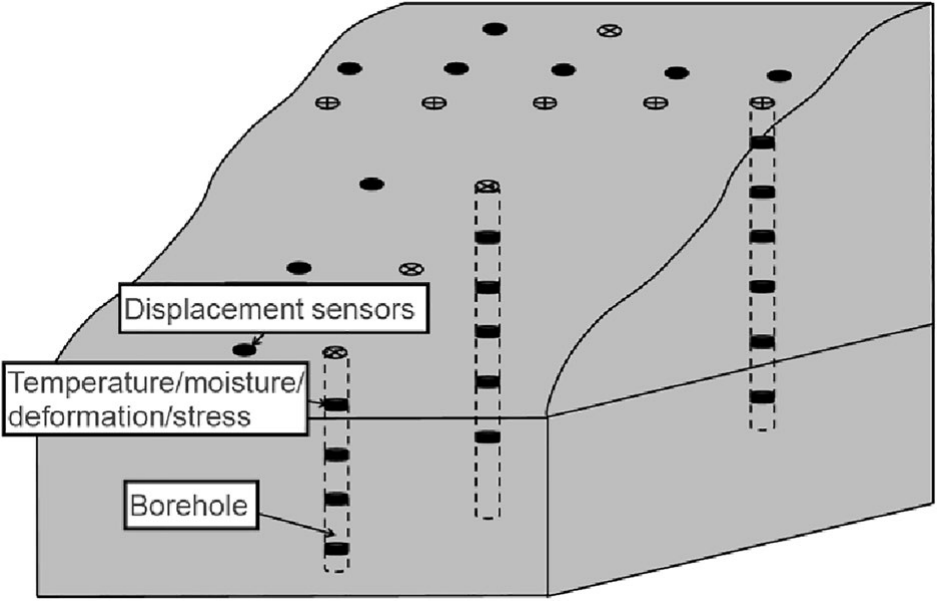
The schematic diagram of field monitoring.

The study of rock slope in cold area should make full use of the actual engineering conditions, and collect the data of temperature, water, stress and deformation of slope extensively, so as to provide basic data for theoretical research and reference for similar engineering construction. The stability of rock slope in cold area is significantly affected by the reciprocating temperature load, therefore, it is necessary to strengthen the monitoring of the temperature field in the field monitoring design, which can reveal the temporal and spatial distribution characteristics of the temperature field of the fractured rock slope under freeze–thaw conditions. In the field monitoring design, the specific installation position of each sensor (temperature, water, stress, deformation) should be determined according to drilling data and indoor test results, and then establish a three-dimensional monitoring network, as shown in Fig. 7.

The study of slope stability in cold region should pay special attention to the occurrence condition of groundwater, which affects the freeze–thaw of slope. However, groundwater measurement is very difficult, especially when there is phase change effect. Therefore, in this paper, it is recommended to use high-precision geological radar to detect the water distribution inside the fractured rock mass slope, so as to understand the migration law and dynamic change of water inside the slope body. Geological radar is the geophysical engineering method with the highest resolution in geological prediction currently used, and its detection principle is based on the special properties such as reflection, refraction and transmission of high-frequency electromagnetic waves in the formation. Due to the filler, water and void in the poor formation, it has a very large dielectric constant difference from the surrounding formation. Therefore, geological radar has a strong ability to identify fractured zones and high aquifers^63^.

## Stability evaluation of fractured rock slope in cold region

The stability evaluation of the frozen rock mass slope is necessary to consider the effects of the influence of freeze–thaw cycles on the rock mass. Currently, however, it is simplified by reducing the parameters of the rock mass in the stability evaluation for such slopes which induced poor reliability. To ensure the reliability of the stability evaluation, it is necessary to consider: (1) The influence of freeze–thaw cycles on the rock parameters, (2) The maximum seepage field on the slope in the warmest season, (3) The maximum frost heave pressure and joint extension.

**Figure 8:**
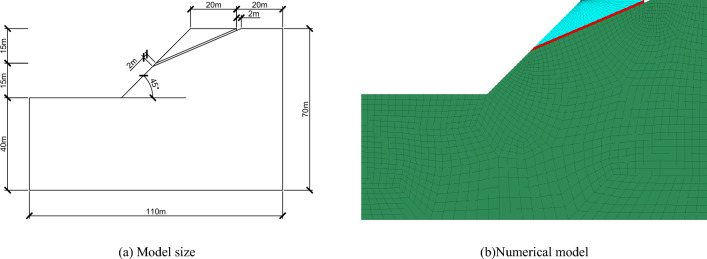
The numerical model for an idealized slope.

In order to demonstrate the influence of the above factors on the stability of rock slope in cold region, the THM coupling theory and program established by Liu et al.^54^ are used to analyze the stability of an ideal slope. The ideal slope is located in the hinterland of the Tibetan Plateau in China. The climate is very cold, and the temperature difference between day and night is large. The annual average air temperature is − 4.2 ℃ to − 5.1 ℃^54^. The numerical model is also generalized from Liu's^54^ paper (see Fig. 8). To simplify the analysis process, only one fracture was created in the model. The boundary conditions and parameters are the same as those in Liu's^54^ paper. In the numerical model, the dark green indicates potential slip body, red indicates fractures, and blue indicates bedrock. In the numerical simulation, the freeze–thaw damage coefficient is introduced to reflect the influence of freeze–thaw cycle; the influence of summer rainfall is reflected by changing the water content of slope; frost heave expansion of fracture is reflected by adjusting the parameters of fracture element.

### Influence of freeze–thaw cycles on the rock parameters

The temperature difference, cycle numbers and duration of freeze–thaw cycle directly affect the mechanical parameters of rock mass, thus affecting the mechanical response of slope, and finally affecting the overall stability of slope. Some scholars have studied the relationship between rock parameters and the number of freeze–thaw cycles and freeze–thaw temperature, and obtained the function of rock cohesion and internal friction angle and the number of freeze–thaw cycles^64,65^, as

1 $$\left\{ \begin{gathered} c = \frac{{c_{0} + \left( {a + b\vartheta + d\vartheta^{2} } \right)n}}{{1 + 0.17\left( {a + b\vartheta + d\vartheta^{2} } \right)n + \left( {d + 0.01\vartheta } \right)n^{2} }} \hfill \\ \varphi = \frac{{\varphi_{0} + \left( {e + f\vartheta^{2} } \right)n}}{{1 + 0.17\left( {e + f\vartheta^{2} } \right)^{{\frac{ - 1}{g}}} n + f\left( {1 + \vartheta } \right)n^{2} }} \hfill \\ \end{gathered} \right.$$

where *c*_0_ is the initial cohesion of unfrozen rock; *φ*_0_ is the initial internal friction angle of unfrozen rock. *n* is the number of freeze–thaw cycles; *θ* is the freezing temperature; *a*, *b*, *d*, *e*, *f*, and *g* are constants.

**Figure 9:**
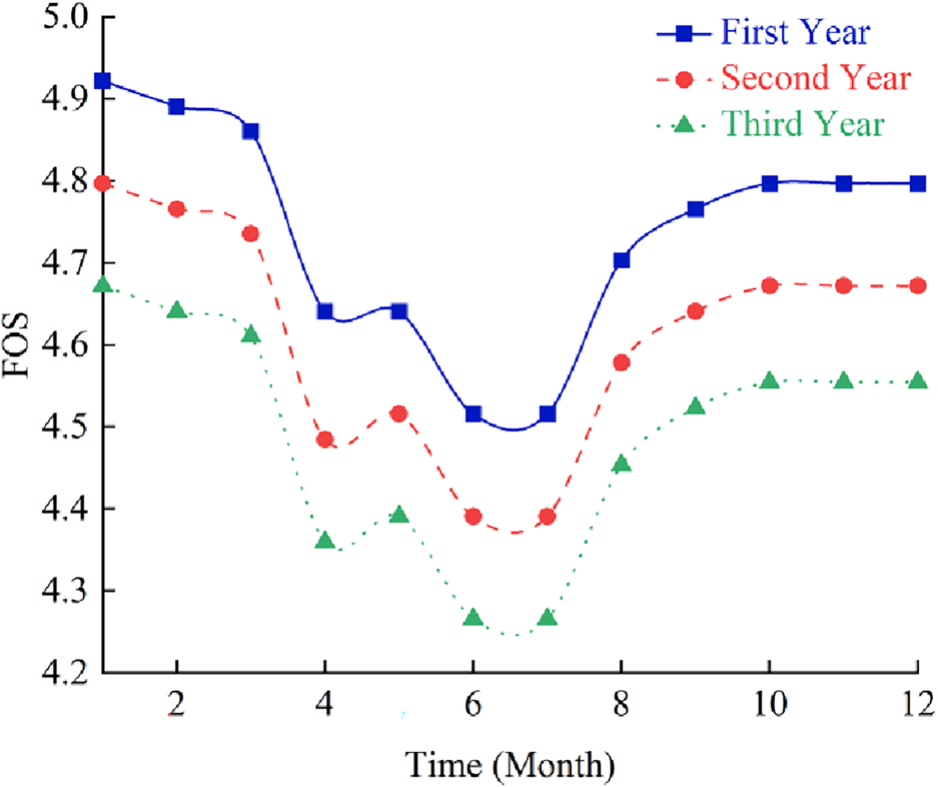
Influence curves of freeze–thaw cycle on safety factor of rock slope in cold regions.

**Figure 10:**
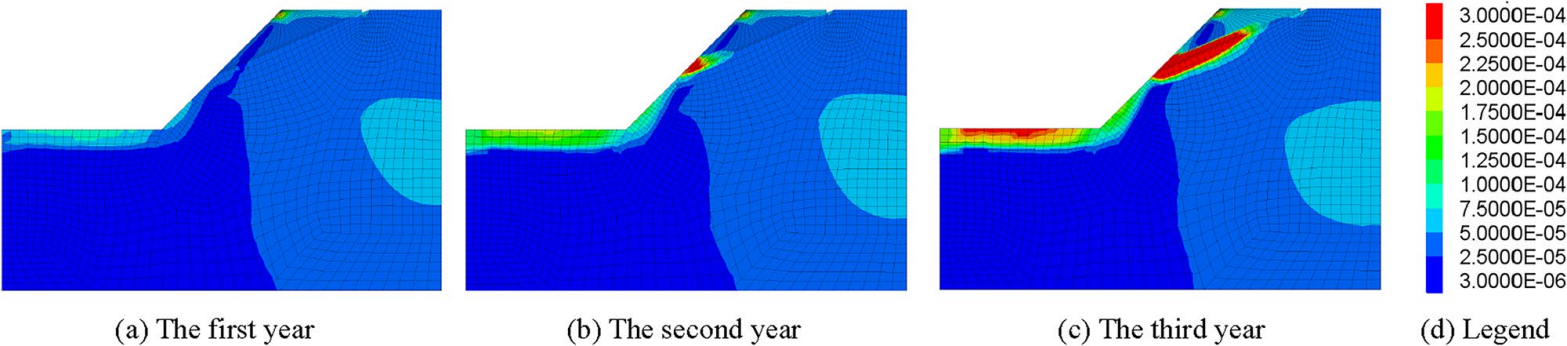
Maximum Shear Strain Increment of slope in December.

Therefore, the influence of freeze–thaw cycle duration on mechanical parameters of rock mass ought to be considered in stability analysis of frozen rock slope. The strength reduction method is used to calculate the safety factor of slope in simulation. After several freeze–thaw cycles, the strength parameter of rock mass decreases, which can be calculated automatically by the program, and the safety factor of slope at this time can also be obtained. The relationship between the slope safety factor (FOS) and freeze–thaw cycle is shown in Fig. 9, and the maximum shear stress increment of the slope at the end of each year is shown in Fig. 10.

It can be seen from Figs. 9 and 10 that the safety factor of frozen rock slope does vary with the freeze–thaw process, rather than keeping constant like ordinary slope. In January, the slope safety factor is the highest. Then, with the gradual warming of the climate, the ice in the rock mass melts, and the safety factor of the slope gradually decreases. Xu^66^ and Wang^67^ also concluded in the experiments that the strength of rocks increases when frozen and decreases after melting. In summer, the slope safety factor decreases to a minimum during the rainy season. After freezing and thawing in April, the slope gradually drained in May, resulting in a decrease in the water content of the slope and a slight increase in the safety factor. After the rainy season, the safety factor of slope increases gradually with the decrease of temperature. In winter, the rock mass froze again and the safety factor of the slope reached a large value. However, due to the deterioration of rock strength caused by freeze–thaw cycle, the slope safety factor in December is less than that in January at the beginning of the year. The change law of safety factor of frozen rock slope in the second and third years is similar to that in the first year. However, due to the freeze–thaw damage of rock mass, the safety factor of slope decreases year by year. Therefore, the deterioration of mechanical parameters of rock mass due to freeze–thaw cycles should be considered in the stability analysis of frozen rock slope.

### The maximum seepage field within the slope in the warmest season

**Figure 11:**
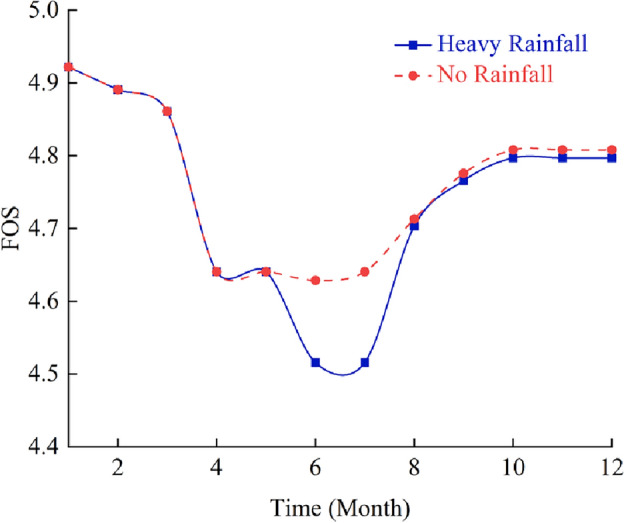
Influence curves of heavy rainfall on slope safety factor.

**Figure 12:**
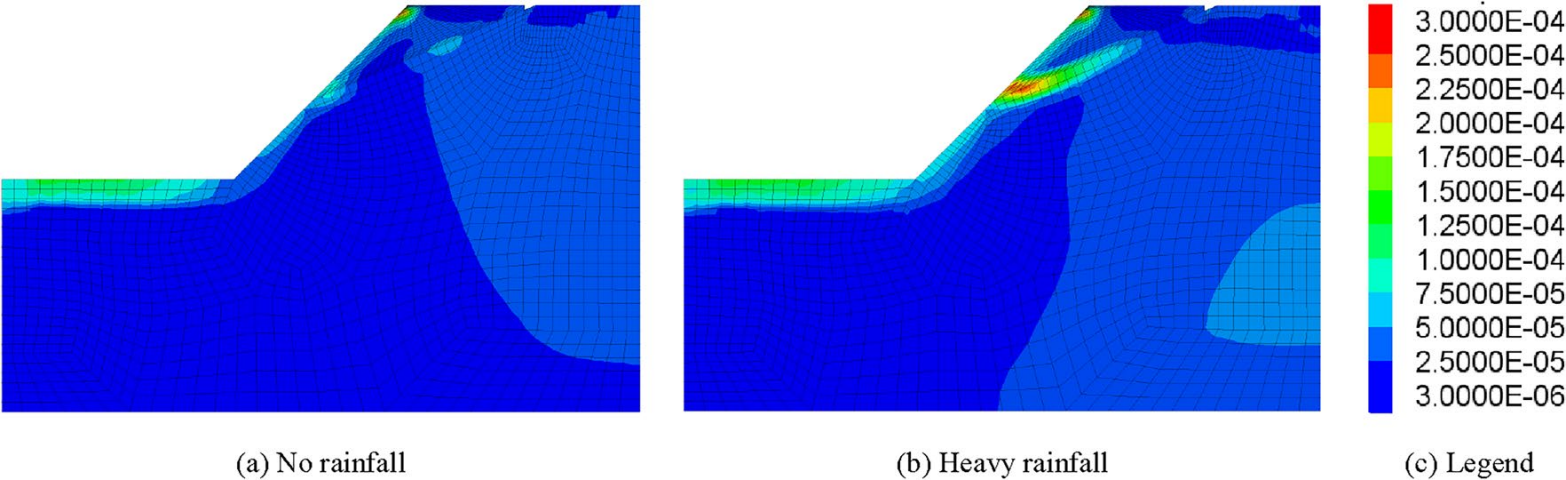
Maximum Shear Strain Increment of slope in July.

Rainfall infiltration has a great influence on slope stability^68,69^, which is mainly manifested as increasing sliding force and reducing sliding resistance, thus affecting the overall stability of the slope. Therefore, the influence of seepage field produced by rainfall in summer on the stability of rock slope in cold area should be considered as well. In order to further confirm the above statement, this section studies the stability of fractured rock slope under the condition of heavy rainfall in summer and compares it with the condition without rainfall. The simulation results are shown in Figs. 11 and 12.

It can be seen from Figs. 11 and 12 that strong rainfall in summer has significant influence on the stability of rock mass slope in cold regions. If the influence of summer rainfall is not considered, the safety factor of rock slope in cold area keeps approximately constant in warm season. However, when the strong rainfall in summer is considered, the safety factor of rock slope in cold area decreases sharply. Therefore, for the stability analysis of rock slope in cold area, not only the collapse damage caused by frost heaving in winter and hot melt slump in spring but also the slope instability caused by strong rainfall seepage field in summer should be considered.

### The maximum frost heaving pressure and fracture expansion

**Figure 13:**
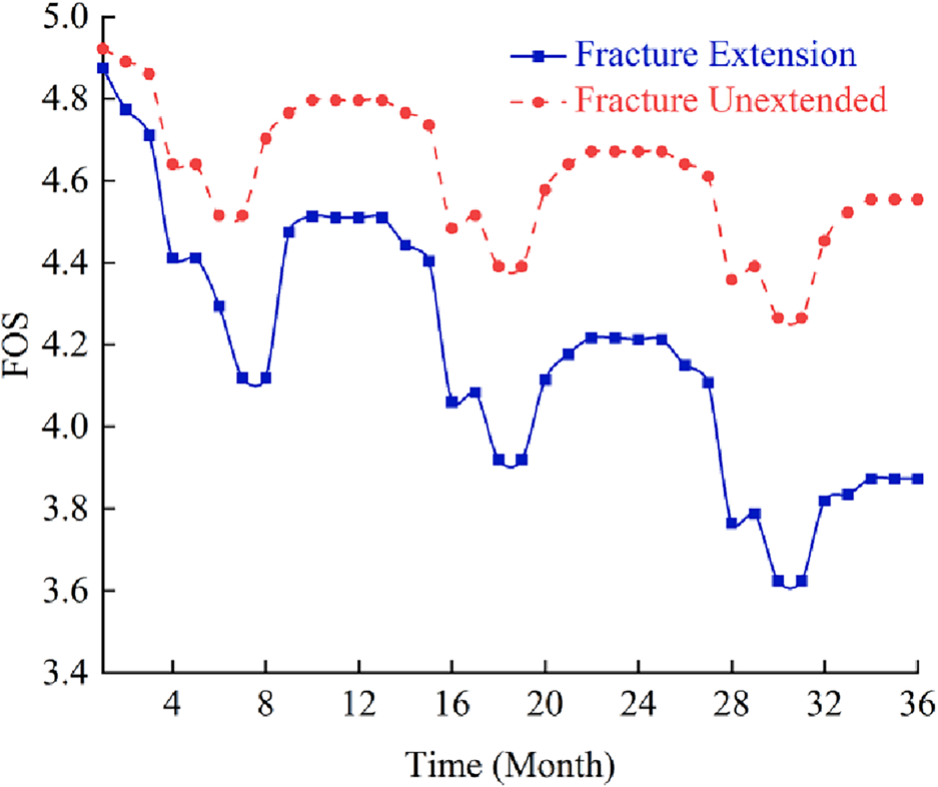
Curves of slope safety factor process under fracture extension and non-extension conditions.

**Figure 14:**
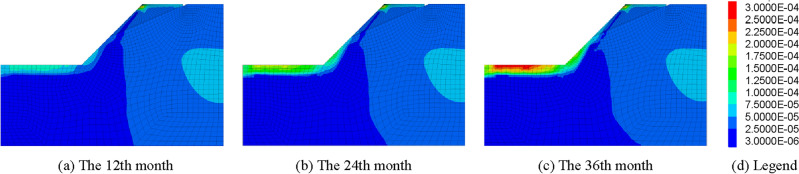
Maximum shear strain increment of slope without fracture extension.

**Figure 15:**
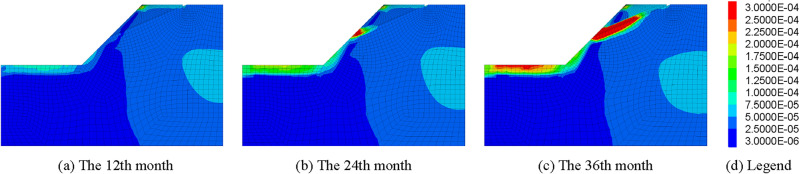
Maximum shear strain increment of slope with fracture extension.

When the frost heaving pressure exceeds the tensile strength of the rock mass, the fracture will be initiated and expanded, thus causing unrecoverable damage to the rock mass. The existence of frost heave force and the expansion of fractures will cause stress concentration in the rock mass of the slope, which will inevitably affect the stability of the rock slope in the cold area. The expanding fractures may even be connected with each other, which may cause geological disasters such as collapse and landslide in serious cases. In order to verify the above, in this section, the stability of rock slopes with fracture extension induced by frost heave force is studied. The stability of frozen rock slope considering fracture extension and no fracture extension is shown in Figs. 13, 14, and 15.

It can be seen from Figs. 13, 14, and 15 that the stability change trends of rock mass slopes in cold regions is basically the same when fracture expansion is considered or not. The safety factor of the slopes is relatively low in summer and relatively high in winter. However, there is also a significant difference in the safety factor of the slope under the two conditions, that is, the decline rate of the safety factor of the frozen rock slope in the spring thaw period is significantly greater than that without considering the fracture expansion. This is because the expansion of fractures due to frost heave damages the integrity of the slope rock mass, reduces the physical properties of the rock mass, improves the water conductivity of the rock mass, and thus affects the stability of the slope. The fracture propagation induced by the water–ice phase transition in winter has a significant impact on the stability of rock slope in cold regions. If the actual existing fracture propagation phenomenon is ignored, the results of slope stability analysis will become more and more inaccurate with the increase of freeze–thaw cycles. Therefore, when evaluating the stability of fractured rock slopes in cold regions, the effect of fracture expansion caused by frost heaving of fracture water should be considered.

### Frozen rock slope stability solutions

The change of slope stability is the result of mutual coupling and dynamic adjustment of moisture field, stress field and temperature field in slope body. The temperature-stress field caused by freeze–thaw cycle and the seepage-stress field caused by the saturation of precipitation in the warmest season have significant effects on the stability of rock slope in cold area. Therefore, it is necessary to establish a new method to evaluate the stability of rock slope in cold region based on the coupling theory of heat-water-force. Since the frozen rock slope is subject to periodic temperature load, so its safety factor can be expressed as a function of time (*t*), temperature (*T*) and water content (*w*), namely:

2 $${\text{FOS}} = F\left( {t,T,w} \right)$$

If the stability of fractured rock slopes under freeze–thaw cycles does not meet the required criteria, freeze–thaw disasters may occur. The study area has witnessed numerous incidents of rock slope collapses associated with freeze–thaw cycles. On April 9, 2000, a catastrophic landslide took place in Bomi County, Nyingchi Prefecture, Xizang Province, resulting in the entrapment of over 4000 individuals and claiming the lives of seven soldiers during rescue operations. These tragic landslide cases highlight that fractured rock slopes are highly susceptible to failure after ice and snow melt in warmer seasons; therefore, it is imperative to implement necessary reinforcement measures. The commonly employed mitigation strategies include: (1) grouting anchoring measures; (2) insulation and anti-freezing techniques; and (3) construction of drainage facilities. Grouting anchoring exhibits effective mitigation results but poses environmental concerns. Insulation and anti-freezing methods can prevent freeze–thaw disasters but suffer from poor economic feasibility for large-scale implementation and struggle to address unstable slopes adequately. Structural drainage measures are often utilized as supplementary approaches^70^. Depending on specific circumstances, these three measures demonstrate significant mitigation effects individually or even when combined. According to different situations, these three measures have a good mitigation effect, and even adopt multiple measures at the same time when necessary.

## Conclusion

In this research, the stability of fractured rock mass slope under freeze–thaw cycles was preliminarily discussed. The main conclusions are as follows.

1. The failure mechanism of frozen rock slope is revealed, and the failure type of frozen rock slope is preliminarily given. The failure forms of frozen rock slope are divided into four types: freeze–thaw collapse of loose mass, freeze–thaw toppling failure, thermal melt collapse failure and deep failure due to frost heaving.
2. The characteristics of frozen fractured rock mass are analyzed, and the engineering classification of frozen rock slope is carried out. Frozen rock slope can be divided into the following four categories: frozen cataclasite slope, frozen blocky rock mass slope, frozen stratified rock mass slope and ice slope containing rocks.
3. A new method for stability evaluation of rock slope in cold region based on THM coupling theory is proposed and verified by numerical simulation. The safety factor of rock slope under freeze–thaw cycle can be expressed as a function of time, temperature and water content.

In this paper, the classification of frozen rock slope and its failure mode is given through investigation, which may be inaccurate and incomplete. In addition, the stability analysis method of fractured rock slope under freeze–thaw cycle is based on simple simulation of ideal slope. While qualitative knowledge is correct, quantitative data may be inaccurate. Therefore, in order to further understand the stability of frozen rock slopes, it is necessary to carry out more comprehensive field investigation, theoretical research and numerical simulation, and develop a software that truly considers the influence of thermo-hydro-mechanical and time, so as to ensure the safe construction of projects in cold areas.

## Data Availability

The data used to support the findings of this study are available from the corresponding author upon request.
